# Barriers and Implications of 5G Technology Adoption for Hospitals in Western China: Integrated Interpretive Structural Modeling and Decision-Making Trial and Evaluation Laboratory Analysis

**DOI:** 10.2196/48842

**Published:** 2024-01-23

**Authors:** Linyun Zhou, Minghuan Jiang, Ran Duan, Feng Zuo, Zongfang Li, Songhua Xu

**Affiliations:** 1 Department of Health Management The Second Affiliated Hospital of Xi’an Jiaotong University Xi’an China; 2 Institute of Medical Artificial Intelligence The Second Affiliated Hospital of Xi’an Jiaotong University Xi’an China; 3 Department of Pharmacy Administration and Clinical Pharmacy School of Pharmacy Xi’an Jiaotong University Xi’an China; 4 Information Department Chongqing General Hospital Chongqing China; 5 Information Center The First Affiliated Hospital of Army Medical University (Southwest Hospital) Chongqing China; 6 Clinical Research Center for Hepatic & Splenic Diseases of Shaanxi Province The Second Affiliated Hospital of Xi’an Jiaotong University Xi'an China

**Keywords:** 5G health care, 5G adoption barriers, 5G adoption strategy, smart health care, Western China hospitals

## Abstract

**Background:**

5G technology is gaining traction in Chinese hospitals for its potential to enhance patient care and internal management. However, various barriers hinder its implementation in clinical settings, and studies on their relevance and importance are scarce.

**Objective:**

This study aimed to identify critical barriers hampering the effective implementation of 5G in hospitals in Western China, to identify interaction relationships and priorities of the above-identified barriers, and to assess the intensity of the relationships and cause-and-effect relations between the adoption barriers.

**Methods:**

This paper uses the Delphi expert consultation method to determine key barriers to 5G adoption in Western China hospitals, the interpretive structural modeling to uncover interaction relationships and priorities, and the decision-making trial and evaluation laboratory method to reveal cause-and-effect relationships and their intensity levels.

**Results:**

In total, 14 barriers were determined by literature review and the Delphi method. Among these, “lack of policies on ethics, rights, and responsibilities in core health care scenarios” emerged as the fundamental influencing factor in the entire system, as it was the only factor at the bottom level of the interpretive structural model. Overall, 8 barriers were classified as the “cause group,” and 6 as the “effect group” by the decision-making trial and evaluation laboratory method. “High expense” and “organizational barriers within hospitals” were determined as the most significant driving barrier (the highest R–C value of 1.361) and the most critical barrier (the highest R+C value of 4.317), respectively.

**Conclusions:**

Promoting the integration of 5G in hospitals in Western China faces multiple complex and interrelated barriers. The study provides valuable quantitative evidence and a comprehensive approach for regulatory authorities, hospitals, and telecom operators, helping them develop strategic pathways for promoting widespread 5G adoption in health care. It is suggested that the stakeholders cooperate to explore and solve the problems in the 5G medical care era, aiming to achieve the coverage of 5G medical care across the country. To our best knowledge, this study is the first academic exploration systematically analyzing factors resisting 5G integration in Chinese hospitals, and it may give subsequent researchers a solid foundation for further studying the application and development of 5G in health care.

## Introduction

### Background

With the advancement of information and communication technology, along with the gradual improvement of China's medical information system construction, China's medical industry is moving away from 1.0 medical informatization to 3.0 medical intelligence [1]. Leveraging the advantages such as ultralow latency, high capacity, high speed, seamless connectivity, high reliability, and low power consumption [2], 5G technology plays an essential role in realizing the interconnection and remote monitoring of medical equipment, patient monitoring, remote consultation, and other telemedicine scenarios. At the same time, 5G technology accelerates the data collection, circulation, analysis, and feedback of various applications in the broad medical and health field. With the advent of 5G technology, medical information can now flow and be shared seamlessly among patients, medical equipment, and hospital information systems. This has paved the way for hospitals to simplify the entire medical treatment and service process, right from prediagnosis to diagnosis and postdiagnosis stages [3].

In recent years, the Chinese government has invested considerably in developing innovative 5G smart hospitals to offer better health care to patients and improve their internal management. For example, in July 2021, a total of 10 departments including the Ministry of Industry and Information Technology, Office of the Central Cyberspace Affairs Commission, National Development and Reform Commission, Ministry of Education, Ministry of Finance, Ministry of Housing and Urban-Rural Development, Ministry of Culture and Tourism, National Health Commission, State-Owned Assets Supervision and Administration Commission of the State Council, and National Energy Administration, jointly released the Sailing Action Plan for 5G Applications (2021-2023). The plan aims to encourage the development of various 5G medical products such as robots, emergency vehicles, medical access gateways, and intelligent medical equipment across the country. The plan also emphasizes the need to strengthen the deployment of 5G medical and health network infrastructure, focusing on improving the coverage of 5G in top-tier national hospitals, disease prevention and control centers, elderly care institutions, and other critical locations. Additionally, the plan aims to build 5G networks and 5G medical edge clouds to enhance in-hospital medical care and telemedicine [4]. In September 2021, the Ministry of Industry and Information Technology, in collaboration with the National Health Commission, released the “Notice on Announcing Pilot Projects for 5G+Medical and Healthcare Applications.” This announcement identified 988 pilot projects aimed at advancing the application of 5G technology in various health care domains, including first aid, telediagnosis, teletreatment, tele-intensive care, traditional Chinese medicine diagnosis and treatment, hospital management, intelligent disease control, health management, and other directions. Among the pilot projects, as many as 611 5G smart medical projects are led by hospitals (general hospitals, emergency centers, and specialized hospitals) [5].

More and more hospitals in China are investing in 5G construction. For instance, Guangdong Provincial People's Hospital put into use the 5G hospital in July 2021, aimed to integrate 5G, big data, artificial intelligence, and other new technologies into various medical scenarios such as treatment, teaching, research, management, and service [6]. Shanghai aims to realize 100% 5G in-depth coverage and 5G typical services for all tertiary hospitals and at least 50% 5G in-depth coverage and 5G typical services for other hospitals by 2023 [7]. Sir Run Run Shaw Hospital Affiliated with Zhejiang University School of Medicine successfully performed a cholecystectomy for a patient from Xinjiang Corps Alar Hospital thousands of miles away by leveraging the robotic arm, which achieved a breakthrough in China's 5G ultraremote robot human liver and gallbladder surgery [8]. The Second Affiliated Hospital of Xi'an Jiaotong University has piloted several scenarios, such as 5G+emergency rescue, 5G+mobile computed tomography, 5G+unmanned aerial vehicle medical delivery, and 5G+integrated remote diagnosis.

However, though 5G technology undoubtedly introduces enormous benefits for hospitals if adequately applied, it has yet to be widely used in many health care scenarios. Hospitals are experiencing various challenges during the actual 5G application process. Different problems are met in the implementation process, including expertise, operation, resource, regulation, and market access factors, as described in the innovation resistance theory (IRT) [9]. At the same time, there are still no systematic studies that have explored the barriers to the adoption of 5G applications in hospitals in Western China. This is particularly important given that technological development in the Eastern region of China is more advanced compared to the Western region. The lag in 5G development in Western China may become another factor that increases the economic imbalance between these 2 regions [10]. Hence, more research is essential for Western China to provide a better understanding of the barriers hindering the adoption of 5G in health care.

### Objectives

This study addresses the critical research question below: what are the barriers to implementing 5G in hospitals in Western China? Based on the question, the following research objectives have been formulated: (1) to identify critical barriers hampering the effective implementation of 5G in hospitals in Western China, (2) to identify interaction relationships and priorities of the above-identified barriers, and (3) to assess the intensity of the relationships and cause-and-effect relations between the adoption barriers.

## Methods

### Ethical Considerations

The data were collected through literature review and anonymous questionnaires, which posed no harm to individuals and did not involve sensitive personal information or commercial interests. Based on the Regulation for Ethical Review of Life Sciences and Medical Research involving human beings issued by the Chinese National Health Commission, Ministry of Education, Ministry of Science and Technology, and State Administration of Traditional Chinese Medicine (Chapter III, Article 32) [11], ethical review was exempted for this study.

### Study Design

**Figure 1:**
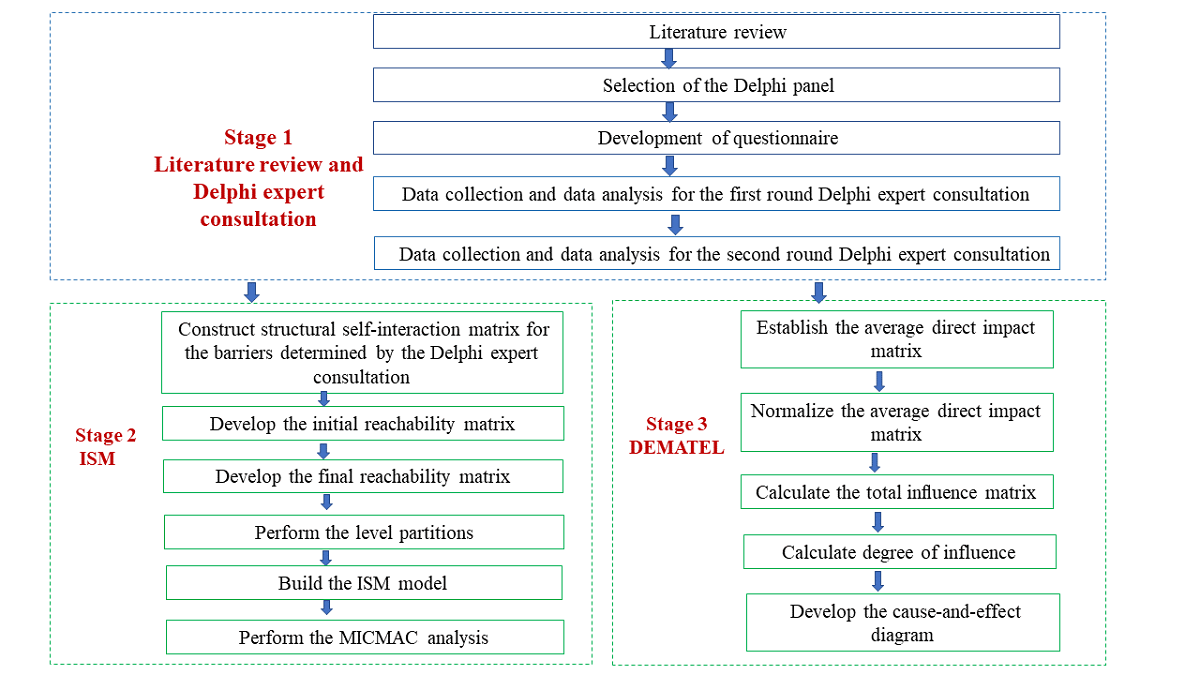
Flowchart of research methodology. DEMATEL: decision-making trial and evaluation laboratory; ISM: interpretive structural modeling; MICMAC: Matriced Impacts Corises-multiplication Appliance Classement.

To address objectives 1-3, a 3-stage hybrid research methodology was proposed by the authors. As shown in Figure 1, a flowchart of the research procedure is conducted. In the first stage, barriers are identified from the existing literature and discussed with experts for further modification and addition using the Delphi technique. The second stage incorporated the interpretive structural modeling (ISM) to obtain a hierarchical structure and interrelationship between the barriers. ISM has a significant advantage because it displays conclusions in the form of a hierarchical topology diagram that is highly intuitive. The hierarchical topology diagram clearly explains the causal relationship and ladder structure among system factors. However, more is needed to determine the intensity of the relationship between factors. It needs to provide the cause-and-effect relation among barriers, which limits the ISM approach [12]. The decision-making trial and evaluation laboratory (DEMATEL) method, on the other hand, can precisely overcome the limitations of the ISM approach. It can determine the strength of influence between variables within the identified structure, providing a deeper understanding of the causal relationship between influencing factors [13]. Therefore, this paper intends to combine Delphi expert consultation, ISM, and DEMATEL to study the hierarchical structure of driving factors and the causal relationship between them. The procedure is explained in more detail in the following section.

### Stage I: Determination of the Barriers Using Literature Review and Delphi Expert Consultation

#### Overview

This study conducted a literature review from November 20 to 30, 2022, to gather the resistance factors toward 5G development in health care. After that, a 2-round Delphi expert consultation was implemented to refine the factors identified from the above literature review on December 30, 2022, and February 9, 2023. The Delphi technique was developed by the Rand Corporation in 1953 and used as a multistage self-completed questionnaire with individual feedback [14]. It was initially developed as a method for forecasting but has since been widely applied in other areas, including health research [15,16].

#### Step 1: Literature Review

Web of Science, PubMed, Google Scholar, Chinese government's official websites, and gray literature, including industry reports, were searched by referencing keywords including “5G healthcare,” “5G smart healthcare,” “5G in hospitals,” “5G applications in healthcare,” and “digital health in China.”

#### Step 2: Selection of the Delphi Panel

To ensure the authoritative scoring results of the consulting expert group on the evaluation indicators, experienced professionals with intermediate or senior titles who are willing to cooperate actively and who are interested in this research were selected. Leaders responsible for informatization work in health authority, heads and frontline employees from the hospital's information management department, 5G communication suppliers, and scholars in 5G and hospital informatization field were invited to this panel.

#### Step 3: Development of Questionnaire

To determine the importance of the barriers selected from the literature review, consolidated criteria have been designed in questionnaire format following a 5-point Likert-type scale (5=very important, 4=relatively important, 3=intermediate, 2=unimportant, and 1=very unimportant). To determine the degree of expert authority (Cr), the expert's familiarity with the indicator (Cs) and the judgment basis (Ca) were collected in the questionnaire. The quantitative values for Cs are divided into 5 levels (0.9=very familiar, 0.7=relatively familiar, 0.5=intermediate, 0.3=unfamiliar, and 0.1=very unfamiliar), and the quantitative values for Ca are present in Table 1. The questionnaire is also designed to allow the experts to offer their judgments, with space provided for them to add, remove, and justify their responses.

**Table 1:** Quantitative values for judgment basis.

Judgment basis (Ca)	Quantitative value of influence degree		
	High	Intermediate	Low
Theoretical analysis	0.3	0.2	0.1
Practical experience	0.5	0.4	0.3
Learn from domestic and foreign peers	0.1	0.1	0.1
Intuition	0.1	0.1	0.1

#### Step 4: Data Collection

The questionnaires were distributed and collected via the WeChat platform, the most widely and frequently used mobile social media in China, which is profoundly integrated into the daily life of Chinese people [17]. It is often used for distributing and collecting questionnaires.

#### Step 5: Data Analysis

An analytical stage followed each round of the Delphi questionnaires. The questionnaire recall rate expresses the degree of positivity of the experts. The degree of expert authority Cr can be calculated from the values of Cs and Ca as follows: Cr=(Cs+Ca)/2. The degree of coordination of expert opinions is judged by the coefficients of variation (CVs) and Kendall coefficient of concordance (W). In this study, the barrier screening standard is CV≤0.250. Barriers whose CVs are higher than 0.250 will be modified or deleted. CV is calculated by the mean value and SD. For Kendall coefficient of concordance, the larger the value, the better the coordination of expert opinions. After the analysis, experts' feedback and perspectives will be presented to all participants.

### Stage II: Development of Research Framework Using ISM

The ISM method originated from structural modeling and was introduced by Warfield [18] for better decision-making when too many factors or constructs exist. It is a qualitative and interpretive method that involves a mutual learning process that uses the experience of experts to identify the relationship between factors, variables, enablers, and barriers [19,20]. Based on the relationship, an overall multilevel structure is extracted from the complex items. It is very suitable for interdisciplinary research of natural science and social science. The ISM method has been widely used in management and new technology research in different industries.

Referring to the above studies, the basic steps of the ISM method in this study are as follows.

Step 1 involved constructing a “structural self-interaction matrix (SSIM)” for the barriers determined by the Delphi expert consultation. In this step, the symbols “L, M, N, and O” are used to develop a link between the proposed barriers, where L indicates that barrier i has an impact on barrier j, M indicates that barrier j has an impact on barrier i, N indicates that barriers i and j interact with each other, and O indicates that barriers i and j have no interaction with each other.

Step 2 involved converting the SSIM into an “initial reachability matrix (IRM).” In this step, the symbols “L, M, N, and O” are converted into binary elements 0 and 1, and the conversion rules are shown in Table 2.

Step 3 involved checking the transitivity of the IRM to obtain the “final reachability matrix (FRM).” Some new interrelationships between barriers can be established during this step. Transitivity was tested as if barrier A inﬂuences barrier B, barrier B inﬂuences barrier C, and then, barrier A indirectly inﬂuences barrier C.

Step 4 involved performing the level partition through the FRM to get the hierarchy of barriers to plot the ISM. Based on the FRM, a “reachability set,” an “antecedent set,” and an “intersection set” for each barrier were developed.

Step 5 involved building the ISM, checking the model for conceptual inconsistencies, and modifying it accordingly.

Step 6 involved performing the Matriced Impacts Corises-multiplication Appliance Classement (MICMAC) analysis. The driving power (DP) and dependence power (DEP) of the identified barriers based on the FRM were calculated, and the barriers were classified into 4 clusters, known as an autonomous cluster, dependent cluster, linkage cluster, and independent cluster. The details of these 4 clusters are the following:

- Autonomous clusters: the barriers within the autonomous cluster have low DEP and DP. These barriers have no direct relation with other barriers and can be considered almost isolated from the system.
- Dependent clusters: the barriers in this group do not have robust DP, but their DEP is strong.
- Linkage clusters: the barriers in this cluster are categorized by high DP and DEP. These factors are unstable, so making any changes to them will significantly affect other barriers and may influence them.
- Independent clusters: the barriers within this cluster have high DP and low DEP. These barriers affect other barriers but are less affected.

**Table 2:** Conversion rule for IRM^a^.

(i,j) in SSIM^b^	(i,j) in IRM	(j,i) in IRM
L^c^	1	0
M^d^	0	1
N^e^	1	1
O^f^	0	0

^a^IRM: initial reachability matrix.

^b^SSIM: structural self-interaction matrix.

^c^L indicates that barrier i has an impact on barrier j.

^d^M indicates that barrier j has an impact on barrier i.

^e^N indicates that barriers i and j interact with each other.

^f^O indicates that barriers i and j have no interaction with each other.

### Stage III: Identification of Cause-and-Effect Group Using DEMATEL

The DEMATEL approach is a system analysis method based on graph theory and matrix tools. It is used to analyze the cause-effect relationship between factors in complex systems and identify the interaction’s intensity [19,21]. The basic steps to carry out DEMATEL analysis are as follows:

Step 1 establishes the average “direct relation matrix.” In this step, experts are invited to evaluate each barrier’s inﬂuence on another using an integer scale. The designed scale has 5 levels, including integers from 0 to 4, where 0 means no impact, 1 means slight impact, 2 means moderate impact, 3 means high impact, and 4 means extremely high impact. Accordingly, the direct influence matrix of each expert is obtained. Then, the average direct relation matrix is obtained by summarizing and averaging all feedback expert data. Given that *k* is the index of experts from a total of *p* experts, *q* is the index of the barriers, and *i* and *j* are the indices for 2 barriers, the decision matrix of each expert is given by 
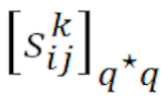
, and then, the direct impact matrix 
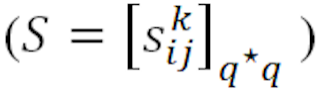
 is given by equation (1).



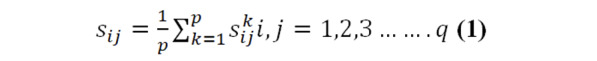



Step 2 normalizes the average direct relation matrix. The row and maximum value methods are used. The elements of each row in the average direct influence matrix are summed, and then, the maximum value is obtained by comparison. Finally, each element in the average direct relation matrix is divided by the maximum value. The calculation process can be expressed as equation (2):


D=S/x **(2)**

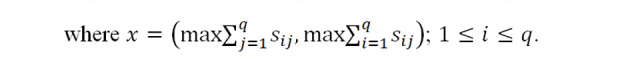



Step 3 calculates the “total inﬂuence matrix T” by adding all the direct and indirect effects using equation (3).



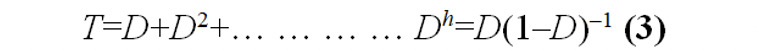



Step 4 develops the “cause-and-effect diagram” by adding elements of vector R (row) and vector C (column) using equations (4) and (5), where, R*_i_* is the sum of the row and C*_j_* is the sum of the column of the “total inﬂuence matrix.” (R*_i_*+C*_j_*) is called the degree of centrality, and (R*_i_*–C*_j_*) is called the degree of cause.



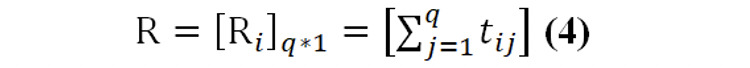





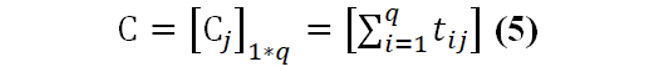



The horizontal and vertical coordinates can be established according to the values of the degrees of centrality and cause. Among them, the degree of centrality is taken as the abscissa, and the degree of cause is taken as the ordinate. Meanwhile, the cause group and effect group are divided according to the positive and negative values of the (R*_i_*–C*_j_*). If the value of (R*_i_*–C*_j_*) is greater than 0, it indicates that this factor has a more significant influence on other factors in the system, and it is classified as a causal factor. If the value of (R*_i_*–C*_j_*) is less than 0, it indicates that other barriers influence this factor greater and attribute it to the outcome factor.

## Results

### Literature Review

The literature review identified 15 factors influencing the adoption of 5G in health care. Based on the IRT, we divided the barriers to adopting innovation into 5 primary dimensions: expertise, operation, resource, regulation, and market access. Compared with the unified theory of acceptance and use of technology, technology acceptance model or technology acceptance model 2, and theory of reasoned action, IRT mentioned above has been verified as an effective and significant alternative for researchers who aim to uncover resistance factors in the health care context [22]. The details are listed in Table 3.

**Table 3:** Barriers influencing adoption of 5G in health care: review of literature.

Barriers		Descriptions
**A. Expertise barrier**		
	A1 Lack of 5G technical talents	5G experts and 5G equipment operators within hospitals are understaffed [23-27].
	A2 Insufficient informatization level	The level of informatization construction of different hospitals is uneven. Significant gaps in equipment networking capabilities, medical data collection, and information integration make it challenging to implement and replicate 5G solutions [28,29].
	A3 Insufficient security verification	Most of the data in the medical field adopt cross-level and multichannel data collection and analysis methods, including hospital management data and private data such as patient physiology, psychology, and behavior data. The security of the data transmitted through 5G network still needs to be verified [28-35].
**B. Operation barrier**		
	B1 Organizational barriers within hospitals	Not a lot of people understand what 5G is and how it works, and the willingness of traditional hospitals to upgrade and transform 5G networks is relatively low, considering the fact that mature 5G application is mainly concentrated in peripheral medical scenarios such as outpatient guidance and remote consultation [25,28,32,36,37].
	B2 Communication obstacles among hospitals	Communication obstacles exist among hospitals, especially among the higher- and lower-level hospitals [24,28,29,38,39].
**C. Resource barrier**		
	C1 High expense	Related equipment and communication costs are high, making it difficult for hospitals to afford [25,30,32,40].
	C2 Huge time cost	Installing appropriate equipment and training relevant personnel demand significant time investment [28,40].
	C3 Lack of well-trained medical and technical personnel	Existing medical care and technical personnel are insufficient for 5G integration in medical scenarios [33].
	C4 Lack of mature compatible equipment and systems	It is difficult for 5G network to integrate with existing equipment and systems [31].
**D.** **Regulation barrier**		
	D1 Lack of policies related to 5G smart medical integration	Currently, there is no established policy for the integration of 5G smart medical applications [24,28,34].
	D2 Lack of ethics, rights, and responsibilities policies	Lack of policies on ethical controversies, rights, and responsibilities related to the application of 5G [28,31,33,34].
	D3 Lack of standards for corresponding scenarios	There are many 5G smart medical application scenarios; different scenarios have different requirements for network and technical architecture. At present, there is a lack of 5G application standards corresponding to many medical scenarios [24,29,41-43].
**E. Market access barrier**		
	E1 Lack of unified 5G product standards and listing standards	Emerging 5G smart medical products (such as wearable intelligent terminal equipment and medical instruments) still need unified and perfect listing standards [28,41].
	E2 Lack of complete 5G smart medical product system	5G private network equipment and terminal equipment that meet the customized services of smart medical care still need to be further improved [28].
	E3 Lack of mature business model	There need to be more mechanisms for cross-field cooperation and mature business models [28].

### Delphi Expert Consultation

The Delphi panel in this study comprises 15 members, including practitioners from the health authority, academia, information management departments of the primary, secondary, and tertiary hospitals, and 5G network operators (see Table 4 for panel composition). A 2-round Delphi expert consultation was conducted to explore the views of different experts on the resistance factors toward 5G development in hospitals. The questionnaire was developed based on the literature review.

In the first and second round, we distributed 15 questionnaires each time. In the first round, all the distributed questionnaires were retrieved, while in the second round, 12 questionnaires were collected. The positive coefficient of experts=number of questionnaires returned/number of questionnaires distributed, which can reflect the degree of concern of experts to this study. Thus, in the first round, the positive coefficient of experts was 100%, while in the second round, this coefficient was reduced to 80%.

In the first and second rounds, we obtained data related to the degree of expert authority as follows:

- Degree of familiarity (Cs): 0.670 in the first round and 0.680 in the second round. It shows that the authority of experts in the 2 rounds of consultation is relatively high, and the opinions given are representative to a certain extent.
- Judgment basis (Ca): 0.930 in the first round and 0.920 in the second round.
- Authority coefficient (Cr): 0.800 for both the first and second rounds.

After the first round of expert consultation, the indicator adjustments are as follows, the selection results can be seen in Table 5:

1. Deleted indicators: original C3 (lack of well-trained medical and technical personnel), original C4 (lack of mature compatible equipment and systems), and original E3 (lack of mature business model).
2. Modified indicators: A1 (lack of personnel familiar with 5G within the hospitals) and D2 (lack of policies on ethics, rights, and responsibilities in core health care scenarios).
3. Newly added indicators: B3 (lack of cross-unit resource integration channels) and new C3 (lack of means for hospitals to manage their own 5G networks).

The consultation process of the second round is consistent with the first round. According to the expert's scoring, the CV was calculated, and the W test was carried out. As can be seen in Table 6, none of the CVs for the second round of barriers were higher than 0.250.

**Figure 2:**
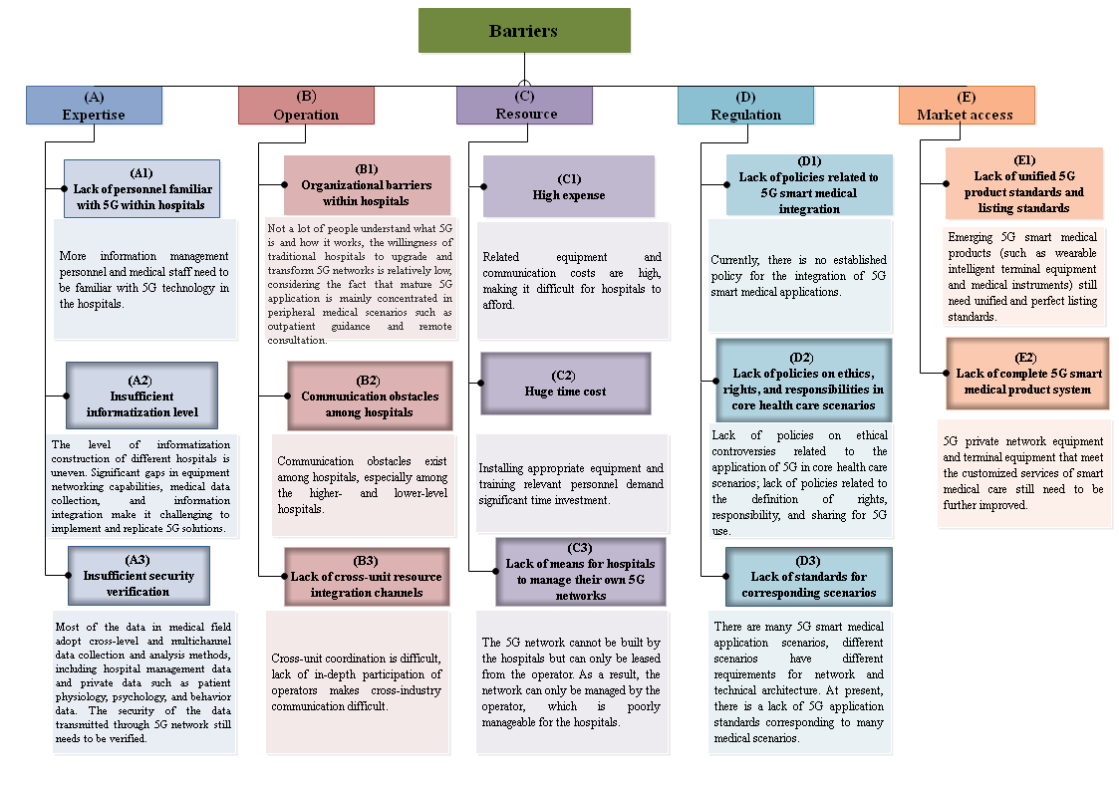
Adoption barriers of 5G for hospitals in Western China.

In the first and second rounds, we obtained the following data on Kendall coefficient of concordance W test: Kendall coefficient of concordance was 0.195 in the first round and 0.258 in the second round, *χ*^2^_14_=40.854 in the first round and *χ*^2^_13_=40.320 in the second round, and *P* value was <.001 for both first and second rounds, which is statistically significant, indicating that the coordination of expert opinions is good. The experts' opinions tended to be unanimous in the second round of consultation, with no modification and new indicators. The final determined barriers can be seen in Figure 2.

**Table 4:** Basic information of Delphi panelist.

Category		Experts, n	Constituent ratio (%)
**Major**			
	Communication technology	2	13
	Computer science and technology	12	80
	Health management	1	7
**Work experience (years)**			
	<10	1	7
	10-19	10	67
	20-29	4	26
**Professional title**			
	Intermediate	1	7
	Vice senior	11	73
	Senior	3	20
**Job description**			
	Information technology operations management	10	67
	Academia	2	13
	Administrative management	3	20

**Table 5:** Selection results of the first round of expert consultation.

	A1	A2	A3	B1	B2	C1	C2	C3	C4	D1	D2	D3	E1	E2	E3
Mean (SD)	3.867 (1.024)	4.267 (0.573)	3.867 (0.806)	4.333 (0.789)	3.200 (0.748)	4.133 (0.884)	3.533 (0.718)	3.267 (1.062)	3.467 (1.087)	3.867 (0.957)	3.467 (0.957)	4.267 (0.573)	4.000 (0.632)	4.133 (0.806)	3.667 (1.247)
CV^a^	0.265	0.134	0.208	0.182	0.234	0.214	0.203	0.325	0.314	0.247	0.276	0.134	0.158	0.195	0.340
Selection criterion (CV≤0.250)	V*^b^	V^c^	V	V	V	V	V	✓^d^	✓	V	V*	V	V	V	✓

^a^CV: coefficient of variation.

^b^The symbol “V*” indicates that the indicator is modified.

^c^The symbol “V” indicates that the indicator is retained.

^d^The symbol “✓“indicates that the indicator is deleted.

**Table 6:** Selection results of the second round of expert consultation.

	A1	A2	A3	B1	B2	B3	C1	C2	C3	D1	D2	D3	E1	E2
Mean (SD)	3.917 (0.640)	4.167 (0.553)	3.917 (0.862)	4.750 (0.433)	3.333 (0.745)	4.250 (0.595)	4.167 (0.799)	3.500 (0.866)	3.333 (0.745)	4.167 (0.898)	3.667 (0.850)	4.083 (0.759)	4.000 (0.707)	4.417 (0.759)
CV^a^	0.163	0.133	0.220	0.091	0.224	0.140	0.192	0.247	0.224	0.215	0.232	0.186	0.177	0.172
Selection criterion (CV≤0.250)	V^b^	V	V	V	V	V	V	V	V	V	V	V	V	V

^a^CV: coefficient of variation.

^b^The symbol “V” indicates that the indicator is retained.

### Results of ISM

#### Establish SSIM

In total, 10 experts from the abovementioned 15 were invited to develop the relationships between different barriers using L, M, N, and O. Due to the nonexistence of a special criterion in the literature of ISM concerning what establishes the majority and as in the methodology of ISM [44], this study adopted a 50% criterion to determine the final relationships between different barriers, such as in a relation, if 5 from 10 experts ticked L, the corresponding column is defined as L. However, to be unbiased, for this kind of relationship, the number of specialists answering by M or N or O should be lesser than or equivalent to 3. Fulfilling both requirements, 47 of 91 cases of relations were included to obtain final results, and the remaining cases were all taken as biased and to be taken for more discussion with experts. To analyze the relations of the remaining barriers, this study proceeded for more discussion with 2 senior experts. To eliminate biases, the selected experts were taken out of the given pool of 10 experts. The 2 senior experts were requested to analyze and discuss the remaining barriers until a consensus was reached. Doing so determined a final relationship between different barriers called SSIM, as shown in Table S1 in Multimedia Appendix 1.

#### Formation of IRM

Transformation of the SSIM into IRM uses the binary rule shown in Table 2. For example, the entry of A1 and D2 in the SSIM is “O,” which is replaced by “0” for A1 and D2 and “0” for D2 and A1 in the IRM (Table S2 in Multimedia Appendix 1).

#### Formation of FRM

FRM is formed after checking IRM for transitivity. It was done to represent all indirect connections to maintain the consistency of relationships among the barriers. For example, there is a direct relation between A1 and C2 and C2 and D1, but there is no relation between A1 and D1, as shown in the SSIM. Hence, according to the transitivity rule, there is an indirect relation between A1 and D1 corrected during the formation of FRM. It can be observed in Table S3 in Multimedia Appendix 1, where the relation of A1 and D1 is represented by 1*. All of the asterisk signs represent the indirect relation rectiﬁed during the formation of the FRM. The FRM calculates each barrier's DP and DEP. The DP is the summation of the value of all the row elements, while the DEP is the summation of all the column elements corresponding to the respective barrier.

#### Level Partition

To have a clearer understanding of the relationship between the barriers, a hierarchical structure of the factors is required. Based on FRM, the reachability set, antecedent set, and intersection set for each barrier were developed. Suppose the reachability and intersection set for a specific barrier are identical. In that case, that barrier is deemed at level 1 and assigned the highest position in the ISM hierarchy. After the first iteration, the barriers constituting level 1 are removed, and the previously mentioned procedure is repeated with the remaining barriers until the levels of all barriers have been determined. The results of the different sets and the level iterations are shown in Table S4 in Multimedia Appendix 1.

#### Formation of ISM

**Figure 3:**
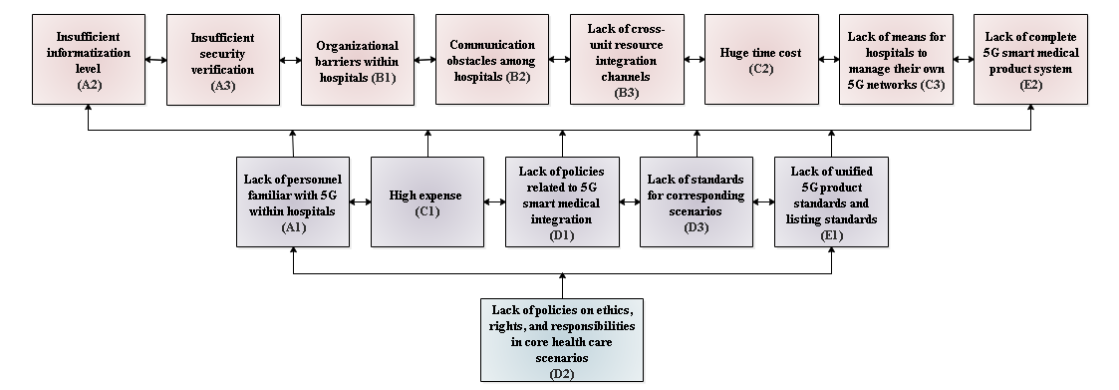
Interpretive structural model.

ISM is formulated based on the partition level of barriers. In the ﬁrst iteration, A2 (insufficient informatization level), A3 (insufficient security verification), B1 (organizational barriers within the hospitals), B2 (communication obstacles among hospitals), B3 (lack of cross-unit resource integration channels), C2 (huge time cost), C3 (lack of means for hospitals to manage their own 5G networks), and E2 (lack of complete 5G smart medical product system) were placed at the top of the ISM. The second iteration resulted in second-level barriers involving A1 (lack of personnel familiar with 5G within hospitals), C1 (high expense), D1 (lack of policies related to 5G smart medical integration), D3 (lack of standards for corresponding scenarios), and E1 (lack of unified 5G product standards and listing standards) placed below the ﬁrst level. Similarly, in the third iteration, D2 (lack of policies on ethics, rights, and responsibilities in core health care scenarios) was placed below the second level. The developed framework or ISM of barrier adoption is shown in Figure 3.

#### MICMAC Analysis

**Figure 4:**
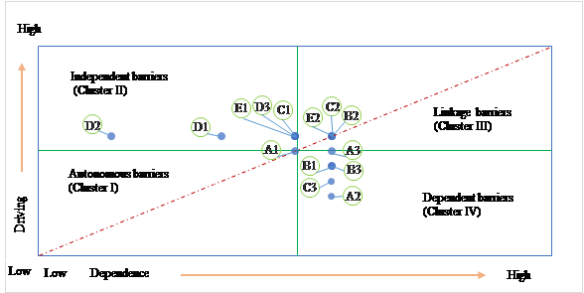
Driving and dependence diagram.

The MICMAC analysis is performed to identify barriers' DP and DEP and classify them accordingly. As shown in Figures 3 and 4, A2 (insufficient informatization level), A3 (insufficient security verification), B1 (organizational barriers within hospitals), B3 (lack of cross-unit resource integration channels), and C3 (lack of means for hospitals to manage their own 5G networks) were placed at the top of the ISM and fell under the “dependent” cluster. B2 (communication obstacles among hospitals), C2 (huge time cost), and E2 (lack of complete 5G smart medical product system) were categorized under the “linkage” cluster. The barriers under the linkage cluster were volatile due to high DP and DEP. C1 (high expense), D1 (lack of policies related to 5G smart medical integration), D2 (lack of policies on ethics, rights, and responsibilities in core health care scenarios), D3 (lack of standards for corresponding scenarios), and E1 (lack of unified 5G product standards and listing standards) were placed in the independent cluster. Considering these barriers as drivers of other barriers in the system, hospitals should prioritize them in their decision-making processes. In addition, D2 (lack of policies on ethics, rights, and responsibilities in core health care scenarios) has a relatively high driving force and low dependence force, which reveals that it strongly impacts the whole system as displayed in the ISM.

### Results of DEMATEL

The abovementioned 10 experts in the ISM scoring process were also invited to participate in the data collection for the DEMATEL analysis. Experts were invited to evaluate each barrier’s inﬂuence on another using a scale of 0-4. After collecting the direct relation matrix of each expert, the average direct relation matrix (Table S5 in Multimedia Appendix 1) was obtained by summarizing and averaging all feedback expert data. Then, the direct relation matrix was converted into a normalized direct relation matrix (Table S6 in Multimedia Appendix 1) using equation (2). Furthermore, the normalized matrix was converted into a total inﬂuence matrix (Table S7 in Multimedia Appendix 1) using equation (3). Finally, the degree of inﬂuence was calculated using equations (4) and (5). The cause-effect matrix is shown in Table S8 in Multimedia Appendix 1.

**Figure 5:**
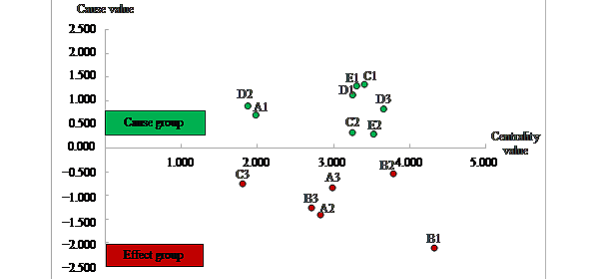
Diagram of cause-effect of barriers.

The barriers with an R–C value less than 0 were identified as the effect group, while barriers with an R–C value greater than 0 fell under the cause group. As shown in Figure 5, a total of 8 barriers could be classiﬁed in the “cause group,” and 6 as the “effect group,” in which C1 (high expense), E1 (lack of unified 5G product standards and listing standards), D1 (lack of policies related to 5G smart medical integration), and D2 (lack of policies on ethics, rights, and responsibilities in core health care scenarios) took high priority in the causal group, B1 (organizational barriers within hospitals) and A2 (insufficient informatization level) were the most inﬂuenced barriers.

### Sensitivity Analysis

**Figure 6:**
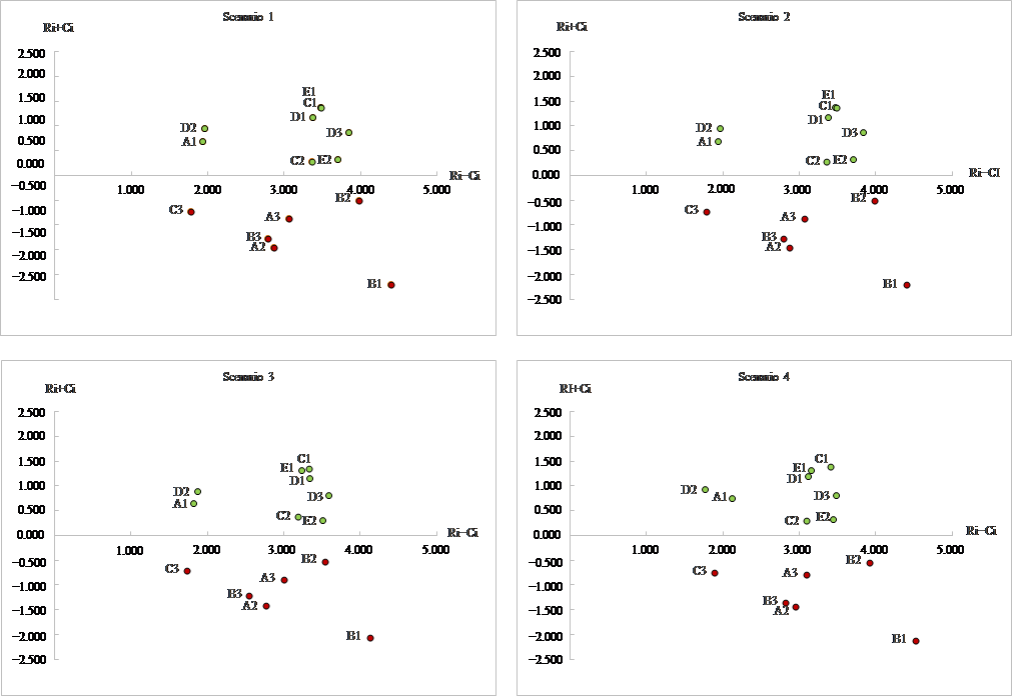
Sensitivity analysis plots.

The sensitivity analysis was performed to verify the consistency of the calculated value and validate the stability of professional judgment. As shown in Table 7, a different weighting was assigned to one expert's feedback while keeping equal weightings for the other. Four different total relationship matrices and comparable matrixes were created by multiplying each weight assigned to the experts, the average relationship matrices were then computed, and the cause-effect relationships among the different barriers were established. As shown in Table 8, the same rank order for cause-effect barriers for each expert was obtained, accepting minor rank order variation. Based on Figure 6, the plots of all barriers during 4 iterations of sensitivity analysis are the same as the base rank. Therefore, it is clear that there was no major change in barrier rankings. The sensitivity analysis conﬁrms the robustness of obtained results.

**Table 7:** Results of sensitivity analysis.

	Scenario 1			Scenario 2			Scenario 3			Scenario 4	
	R+C	R–C	R+C		R–C	R+C		R–C	R+C		R–C
A1	1.939	0.678	1.939		0.678	1.824		0.643	2.115		0.742
A2	2.862	–1.448	2.862		–1.448	2.761		–1.413	2.947		–1.435
A3	3.062	–0.864	3.062		–0.864	3.000		–0.893	3.086		–0.785
B1	4.401	–2.207	4.401		–2.207	4.127		–2.056	4.518		–2.123
B2	3.982	–0.510	3.982		–0.510	3.533		–0.535	3.914		–0.543
B3	2.792	–1.266	2.792		–1.266	2.544		–1.212	2.812		–1.349
C1	3.482	1.372	3.482		1.372	3.329		1.349	3.411		1.383
C2	3.358	0.287	3.358		0.287	3.177		0.387	3.091		0.298
C3	1.780	–0.720	1.780		–0.720	1.724		–0.710	1.879		–0.748
D1	3.379	1.171	3.379		1.171	3.341		1.153	3.120		1.195
D2	1.962	0.955	1.962		0.955	1.876		0.882	1.767		0.921
D3	3.843	0.863	3.843		0.863	3.588		0.811	3.479		0.815
E1	3.474	1.373	3.474		1.373	3.240		1.303	3.162		1.315
E2	3.701	0.316	3.701		0.316	3.504		0.293	3.450		0.315

**Table 8:** Ranking obtained after sensitivity analysis.

	Inputs for sensitivity analysis			
	Scenario 1	Scenario 2	Scenario 3	Scenario 4
Expert 1	0.4	0.2	0.2	0.2
Expert 2	0.2	0.4	0.2	0.2
Expert 3	0.2	0.2	0.4	0.2
Expert 4	0.2	0.2	0.2	0.4

## Discussion

### Principal Results

As shown in Figures 3 and 5, all the barriers in the bottom and middle levels fell into the cause group, indicating those barriers are the primary reasons why 5G technology cannot be adopted in hospitals. In particular, D2 (lack of policies on ethics, rights, and responsibilities in core health care scenarios) was the only factor at the bottom level, implying it is the underlying influencing factor of the whole system. It also has a relatively high driving force and low dependence force based on the MICMAC analysis, which is consistent with the analysis results of DEMATEL as it took high priority in the causal group with an R–C value of 0.900 and an R+C value of 1.875. The finding aligns with that of [28], which highlights that medical and health care fields are closely linked to people's safety. Additionally, ethical considerations and definitions of responsibilities and risks are the most significant obstacles to the evolution and development of 5G in the core areas of health care. It is suggested that the government should strengthen policy supervision to ensure the safe and ethical use of 5G in the medical field. Specific measures need to be implemented to supervise and regulate the application of 5G in the medical field. In addition, further research on relevant policies and norms is required. At the same time, clarification should be made in policy documents, laws, and regulations.

Another significant obstacle in promoting 5G medical applications is the massive capital investment required, as stated in C1, with the highest R–C value of 1.361, indicating its most significant driving force over other barriers. This finding reflects the facts stated by CN-HEALTHCARE [45]. The overall cost of 5G medical treatment includes the construction of 5G communication networks by operators, procurement and maintenance costs for 5G medical information systems and related medical equipment, purchase of medical care equipment, and services for users. The application of 5G in hospitals also requires installing indoor base stations, software support, and computer room upgrades, all of which incur significant expenses. The high cost of 5G applications limits the financing capabilities of hospitals, especially in the Western China region, where hospitals are generally smaller and have limited funding. As a result, only a few large hospitals with telecom operator support have been able to implement 5G medical care. Meanwhile, smaller hospitals are expected to undertake 5G telemedicine with larger tertiary hospitals. Thus, at this stage, it is recommended that smaller hospitals increase their collaboration with larger hospitals to accelerate the adoption of 5G. At the same time, the government is essential to develop affordable solutions and provide financial support for 5G adoption in health care.

With the highest R+C value of 4.317, B1 (organizational barriers within hospitals) is the most closely related barrier to other factors, indicating that it is the most important barrier to adopting 5G in hospitals in Western China. It also has the lowest R–C value of –2.107, which means it is in the effect group and the most influenced barriers by other factors. These results echo the findings of CN-HEALTHCARE [46], which identified a lack of mature 5G applications in medical scenarios, varying levels of acceptance of new technology, concerns about 5G security, and limitations in human, financial, and material resources as factors influencing attitudes toward 5G adoption among different hospitals. Organizational barriers may be addressed through effective management and leadership, clear communication, and collaboration among different departments.

E1 (lack of unified 5G product standards and listing standards) has the second-highest R–C value of 1.316 and a relatively high R+C value of 3.300. The high R–C value specifies its driving force over the other barriers, while the high R+C value indicates its strong impact on the adoption process. This outcome is akin to the findings of Bruer and Doug [47], who mentioned that 5G standards play a crucial role in hardware infrastructure to software running on top of components, and unified 5G standards help to ensure that a range of devices and equipment can operate in a shared system. Therefore, developing unified 5G product standards and listing standards for corresponding scenarios are essential. It is recommended that the Chinese government, 5G network operators, and hospital administrators accelerate cooperation to establish unified 5G product standards and list standards to jointly promote the large-scale development of 5G in the health care sector.

B2 (communication obstacles among hospitals) is a part of the linkage cluster with the second-highest R+C value of 3.782, indicating it is one of the crucial factors in the whole system. This finding is consistent with that of Wang et al [39], who found that effective communication is critical for the success of 5G adoption in health care. Communication obstacles can lead to misunderstandings and a lack of trust. It is urgent to break through the communication obstacles among hospitals, especially between the higher- and lower-level hospitals, which need to accelerate top-level design, formulate policy documents, and improve relevant legislation and management mechanisms to promote the opening and sharing of 5G medical data and ensure the deep integration of the 5G medical industry.

### Concluding Remarks

The paper comprehensively analyzes barriers to 5G adoption in hospitals in Western China. Experts from different stakeholders validated 14 resistance factors. Next, an integrated ISM-DEMATEL approach was applied to model the barriers as a network of factors and alternatives categorized into clusters. All barriers were related to human expertise, resource allocation, operational procedures, laws and regulations, and market access capability. Overall, the study shows that promoting the integration of 5G in hospitals in Western China faces multiple complex and interrelated barriers. It constructs a framework for the main barriers to 5G adoption in the hospital context and provides regulatory authorities, hospital managers, and telecom operators with theoretical and managerial insights into the strategic pathways.

### Theoretical and Managerial Implications

- The barrier at the bottom level of the ISM should be emphasized for short-term strategy. The middle-level barriers can be considered for medium- and long-term strategies. The barriers at the top of the ISM can be a long-term strategic focus.
- The effect group can easily be influenced by the cause group, and therefore, managers should give the most attention to causal barriers when implementing 5G practices in hospitals.
- The ranking of cause-effect group barriers can assist regulatory authorities, hospital managers, and telecom operators in developing strategic policy during 5G implementation.
- To overcome these barriers, hospital managers should formulate a 5G adoption strategy that considers the specific needs of the institution and the local market. Specific measures include increasing investment in information infrastructure and human resources, establishing supplier communication channels, and promoting cross-unit resource integration.
- To expand the use of 5G in health care scenarios, it is recommended that the government accelerates the construction of an innovation system consisting of regulators, hospitals, telecom operators, academic researchers, and patient representatives.

### Outlook of 5G Health Care

As the infrastructure of intelligent medical care, 5G allows the transmission of vast amounts of data and information, making the information superhighway a reality. Furthermore, with the full deployment of 5G medical care, especially the integration with big data, artificial intelligence, internet, internet of things, and blockchain technology, 5G is expected to bring significant changes to the current medical and health system and promote the evolution of the entire medical ecology, including hospital operation and management. In the long run, 5G health care promotes the sinking of high-quality medical resources and the development of China's “primary health care” and “family doctor” systems. It can improve the population's overall health, reduce medical expenses for ordinary people, and relieve medical insurance burdens. Therefore, no matter how barriers are faced, the benefits of 5G medical care are expected to outweigh the costs, making it a worthy investment. In the development of 5G medical care, various technical, economic, institutional, interest, and ethical problems will inevitably be encountered. The regulatory authorities, hospitals, telecom operators, and the public must cooperate to explore and solve the problems in the 5G medical care era, aiming to achieve the coverage of 5G medical care across the country.

### Limitations

Although this study suggests some useful implications, there are some limitations that could be considered for future research. First, due to the challenges in reaching out to health care professionals and telecom operators from all regions of China, most of the experts invited for this research were from representative cities in Northwest and Southwest China, including Xi'an, Chongqing, and Chengdu. This may not fully represent the entire country, as Eastern China is generally more developed than Western China. Therefore, there is a lack of balance among the groups of participants in this research. In future research, we plan to invite experts from Eastern China as a complement study. Second, the relations established among barriers might be biased because they are selected and analyzed based on expert opinions that are context-dependent and depend on their organization’s culture and experience. Third, the outcome of this study is valid for the Chinese health care field and cannot be generalized for other sectors without modiﬁcations. It can be extended from the Chinese context to a broader coverage by selecting experts from different countries for benchmarking studies. Finally, only 4 groups of experts, namely, government information department staff, managers from the hospital information technology departments, telecom operators, and scholars, are involved in the research process. Other vital stakeholders, such as patients, can also provide crucial information and insights related to the development of 5G health care.

## Appendix

Multimedia Appendix 1 Interpretive structural modeling model and decision-making trial and evaluation laboratory method operation process.

## Abbreviations

CV: coefficient of variation
DEMATEL: decision-making trial and evaluation laboratory
DEP: dependence power
DP: driving power
FRM: final reachability matrix
IRM: initial reachability matrix
IRT: innovation resistance theory
ISM: interpretive structural modeling
MICMAC: Matriced Impacts Corises-multiplication Appliance Classement
SSIM: structural self-interaction matrix
