# Supplementary material for: Barriers and Implications of 5G Technology Adoption for Hospitals in Western China: Integrated Interpretive Structural Modeling and Decision-Making Trial and Evaluation Laboratory Analysis
Source: JMIR Mhealth Uhealth. 2024 Jan 23;12:e48842. doi: 10.2196/48842 (PMC10848141; doi:10.2196/48842)
Supplement: Multimedia Appendix 1 [file mhealth_v12i1e48842_app1.docx]

**Multimedia Appendix 1.** Interpretive structural modeling model and decision-making trial and evaluation laboratory method operation process.

**Table S1.** Structural self-interaction matrix (SSIM).

|  | **A1** | **A2** | **A3** | **B1** | **B2** | **B3** | **C1** | **C2** | **C3** | **D1** | **D2** | **D3** | **E1** | **E2** |
| --- | --- | --- | --- | --- | --- | --- | --- | --- | --- | --- | --- | --- | --- | --- |
| **A1** |  | **L** | **L** | **N** | **L** | **L** | **O** | **L** | **L** | **M** | **O** | **O** | **O** | **O** |
| **A2** |  |  | **N** | **N** | **M** | **L** | **M** | **M** | **M** | **O** | **O** | **M** | **O** | **O** |
| **A3** |  |  |  | **L** | **L** | **L** | **M** | **M** | **O** | **M** | **M** | **M** | **M** | **N** |
| **B1** |  |  |  |  | **N** | **L** | **M** | **M** | **N** | **M** | **M** | **M** | **M** | **M** |
| **B2** |  |  |  |  |  | **N** | **M** | **L** | **N** | **M** | **O** | **N** | **N** | **N** |
| **B3** |  |  |  |  |  |  | **N** | **L** | **O** | **M** | **M** | **M** | **O** | **O** |
| **C1** |  |  |  |  |  |  |  | **N** | **L** | **L** | **O** | **L** | **M** | **N** |
| **C2** |  |  |  |  |  |  |  |  | **L** | **L** | **O** | **N** | **N** | **M** |
| **C3** |  |  |  |  |  |  |  |  |  | **M** | **O** | **O** | **O** | **O** |
| **D1** |  |  |  |  |  |  |  |  |  |  | **N** | **N** | **N** | **N** |
| **D2** |  |  |  |  |  |  |  |  |  |  |  | **N** | **N** | **L** |
| **D3** |  |  |  |  |  |  |  |  |  |  |  |  | **N** | **N** |
| **E1** |  |  |  |  |  |  |  |  |  |  |  |  |  | **L** |

**Table** **S2.** Initial reachability matrix.

|  | **A1** | **A2** | **A3** | **B1** | **B2** | **B3** | **C1** | **C2** | **C3** | **D1** | **D2** | **D3** | **E1** | **E2** |
| --- | --- | --- | --- | --- | --- | --- | --- | --- | --- | --- | --- | --- | --- | --- |
| **A1** | **0** | **1** | **1** | **1** | **1** | **1** | **0** | **1** | **1** | **0** | **0** | **0** | **0** | **0** |
| **A2** | **0** | **0** | **1** | **1** | **0** | **1** | **0** | **0** | **0** | **0** | **0** | **0** | **0** | **0** |
| **A3** | **0** | **1** | **0** | **1** | **1** | **1** | **0** | **0** | **0** | **0** | **0** | **0** | **0** | **1** |
| **B1** | **1** | **1** | **0** | **0** | **1** | **1** | **0** | **0** | **1** | **0** | **0** | **0** | **0** | **0** |
| **B2** | **0** | **1** | **0** | **1** | **0** | **1** | **0** | **1** | **1** | **0** | **0** | **1** | **1** | **1** |
| **B3** | **0** | **0** | **0** | **0** | **1** | **0** | **1** | **1** | **0** | **0** | **0** | **0** | **0** | **0** |
| **C1** | **0** | **1** | **1** | **1** | **1** | **1** | **0** | **1** | **1** | **1** | **0** | **1** | **0** | **1** |
| **C2** | **0** | **1** | **1** | **1** | **0** | **0** | **1** | **0** | **1** | **1** | **0** | **1** | **1** | **0** |
| **C3** | **0** | **1** | **0** | **1** | **1** | **0** | **0** | **0** | **0** | **0** | **0** | **0** | **0** | **0** |
| **D1** | **1** | **0** | **1** | **1** | **1** | **1** | **0** | **0** | **1** | **0** | **1** | **1** | **1** | **1** |
| **D2** | **0** | **0** | **1** | **1** | **0** | **1** | **0** | **0** | **0** | **1** | **0** | **1** | **1** | **1** |
| **D3** | **0** | **1** | **1** | **1** | **1** | **1** | **0** | **1** | **0** | **1** | **1** | **0** | **1** | **1** |
| **E1** | **0** | **0** | **1** | **1** | **1** | **0** | **1** | **1** | **0** | **1** | **1** | **1** | **0** | **1** |
| **E2** | **0** | **0** | **1** | **1** | **1** | **0** | **1** | **1** | **0** | **1** | **0** | **1** | **0** | **0** |

**Table S3.** Final reachability matrix.

|  | **A1** | **A2** | **A3** | **B1** | **B2** | **B3** | **C1** | **C2** | **C3** | **D1** | **D2** | **D3** | **E1** | **E2** | **DP** |
| --- | --- | --- | --- | --- | --- | --- | --- | --- | --- | --- | --- | --- | --- | --- | --- |
| **A1** | **1*** | **1** | **1** | **1** | **1** | **1** | **1*** | **1** | **1** | **1*** | **0** | **1*** | **1*** | **1*** | 13 |
| **A2** | **1*** | **1*** | **1** | **1** | **1*** | **1** | **1*** | **1*** | **1*** | **0** | **0** | **0** | **0** | **1*** | 10 |
| **A3** | **1*** | **1** | **1*** | **1** | **1** | **1** | **1*** | **1*** | **1*** | **1*** | **0** | **1*** | **1*** | **1** | 13 |
| **B1** | **1** | **1** | **1*** | **1*** | **1** | **1** | **1*** | **1*** | **1** | **0** | **0** | **1*** | **1*** | **1*** | 12 |
| **B2** | **1*** | **1** | **1*** | **1** | **1*** | **1** | **1*** | **1** | **1** | **1*** | **1*** | **1** | **1** | **1** | **14** |
| **B3** | **0** | **1*** | **1*** | **1*** | **1** | **1*** | **1** | **1** | **1*** | **1*** | **0** | **1*** | **1*** | **1*** | 12 |
| **C1** | **1*** | **1** | **1** | **1** | **1** | **1** | **1*** | **1** | **1** | **1** | **1*** | **1** | **1*** | **1** | **14** |
| **C2** | **1*** | **1** | **1** | **1** | **1*** | **1*** | **1** | **1*** | **1** | **1** | **1*** | **1** | **1** | **1*** | **14** |
| **C3** | **1*** | **1** | **1*** | **1** | **1** | **1*** | **0** | **1*** | **1*** | **0** | **0** | **1*** | **1*** | **1*** | 11 |
| **D1** | **1** | **1*** | **1** | **1** | **1** | **1** | **1*** | **1*** | **1** | **1*** | **1** | **1** | **1** | **1** | **14** |
| **D2** | **1*** | **1*** | **1** | **1** | **1*** | **1** | **1*** | **1*** | **1*** | **1** | **1*** | **1** | **1** | **1** | **14** |
| **D3** | **1*** | **1** | **1** | **1** | **1** | **1** | **1*** | **1** | **1*** | **1** | **1** | **1*** | **1** | **1** | **14** |
| **E1** | **1*** | **1*** | **1** | **1** | **1** | **1*** | **1** | **1** | **1*** | **1** | **1** | **1** | **1*** | **1** | **14** |
| **E2** | **1*** | **1*** | **1** | **1** | **1** | **1*** | **1** | **1** | **1*** | **1** | **1*** | **1** | **1*** | **1*** | **14** |
| **DEP** | 13 | 14 | **14** | **14** | **14** | **14** | **13** | **14** | **14** | **11** | **8** | **13** | **13** | **14** |  |

**Table S4.** Level partition results.

| **Barriers** | **Reachability set** | **Antecedent set** | **Intersection set** | **Level** |
| --- | --- | --- | --- | --- |
| A1 | A1 A2 A3 B1 B2 B3 C1 C2 C3 D1 D3 E1 E2 | A1 A2 A3 B1 B2 C1 C2 C3 D1 D2 D3 E1 E2 | A1 A2 A3 B1 B2 C1 C2 C3 D1 D3 E1 E2 | 2 |
| A2 | A1 A2 A3 B1 B2 C1 C2 C3 E2 | A1 A2 A3 B1 B2 C1 C2 C3 D1 D2 D3 E1 E2 | A1 A2 A3 B1 B2 C1 C2 C3 E2 | 1 |
| A3 | A1 A2 A3 B1 B2 B3 C1 C2 C3 D1 D3 E1 E2 | A1 A2 A3 B1 B2 B3 C1 C2 C3 D1 D2 D3 E1 E2 | A1 A2 A3 B1 B2 B3 C1 C2 C3 D1 D3 E1 E2 | 1 |
| B1 | A1 A2 A3 B1 B2 B3 C1 C2 C3 D3 E1 E2 | A1 A2 A3 B1 B2 B3 C1 C2 C3 D1 D2 D3 E1 E2 | A1 A2 A3 B1 B2 B3 C1 C2 C3 D3 E1 E2 | 1 |
| B2 | A1 A2 A3 B1 B2 B3 C1 C2 C3 D1 D2 D3 E1 E2 | A1 A2 A3 B1 B2 B3 C1 C2 C3 D1 D2 D3 E1 E2 | A1 A2 A3 B1 B2 B3 C1 C2 C3 D1 D2 D3 E1 E2 | 1 |
| B3 | A2 A3 B1 B2 B3 C1 C2 C3 D1 D3 E1 E2 | A1 A2 A3 B1 B2 B3 C1 C2 C3 D1 D2 D3 E1 E2 | A2 A3 B1 B2 B3 C1 C2 C3 D1 D3 E1 E2 | 1 |
| C1 | A1 A2 A3 B1 B2 B3 C1 C2 C3 D1 D2 D3 E1 E2 | A1 A2 A3 B1 B2 B3 C1 C2 D1 D2 D3 E1 E2 | A1 A2 A3 B1 B2 B3 C1 C2 D1 D2 D3 E1 E2 | 2 |
| C2 | A1 A2 A3 B1 B2 B3 C1 C2 C3 D1 D2 D3 E1 E2 | A1 A2 A3 B1 B2 B3 C1 C2 C3 D1 D2 D3 E1 E2 | A1 A2 A3 B1 B2 B3 C1 C2 C3 D1 D2 D3 E1 E2 | 1 |
| C3 | A1 A2 A3 B1 B2 B3 C1 C2 C3 D1 D2 D3 E1 E2 | A1 A2 A3 B1 B2 B3 C1 C2 C3 D1 D2 D3 E1 E2 | A1 A2 A3 B1 B2 B3 C1 C2 C3 D1 D2 D3 E1 E2 | 1 |
| D1 | A1 A2 A3 B1 B2 B3 C1 C2 C3 D1 D2 D3 E1 E2 | A1 A3 B2 B3 C1 C2 D1 D2 D3 E1 E2 | A1 A3 B2 B3 C1 C2 D1 D2 D3 E1 E2 | 2 |
| D2 | A1 A2 A3 B1 B2 B3 C1 C2 C3 D1 D2 D3 E1 E2 | B2 C1 C2 D1 D2 D3 E1 E2 | B2 C1 C2 D1 D2 D3 E1 E2 | 3 |
| D3 | A1 A2 A3 B1 B2 B3 C1 C2 C3 D1 D2 D3 E1 E2 | A1 A3 B1 B2 B3 C1 C2 C3 D1 D2 D3 E1 E2 | A1 A3 B1 B2 B3 C1 C2 C3 D1 D2 D3 E1 E2 | 2 |
| E1 | A1 A2 A3 B1 B2 B3 C1 C2 C3 D1 D2 D3 E1 E2 | A1 A3 B1 B2 B3 C1 C2 C3 D1 D2 D3 E1 E2 | A1 A3 B1 B2 B3 C1 C2 C3 D1 D2 D3 E1 E2 | 2 |

**Table S5.** Direct relation matrix.

|  | A1 | A2 | A3 | B1 | B2 | B3 | C1 | C2 | C3 | D1 | D2 | D3 | E1 | E2 |
| --- | --- | --- | --- | --- | --- | --- | --- | --- | --- | --- | --- | --- | --- | --- |
| A1 | 0.000 | 2.100 | 1.700 | 1.800 | 2.400 | 1.600 | 0.000 | 1.000 | 2.400 | 0.000 | 0.000 | 0.000 | 0.000 | 0.000 |
| A2 | 0.000 | 0.000 | 2.100 | 2.700 | 0.000 | 2.200 | 0.000 | 0.000 | 0.000 | 0.000 | 0.000 | 0.000 | 0.000 | 0.000 |
| A3 | 0.000 | 2.000 | 0.000 | 1.900 | 1.800 | 1.500 | 0.000 | 0.000 | 0.000 | 0.000 | 0.000 | 0.000 | 0.000 | 2.200 |
| B1 | 2.200 | 2.700 | 0.000 | 0.000 | 2.200 | 2.200 | 0.000 | 0.000 | 1.600 | 0.000 | 0.000 | 0.000 | 0.000 | 0.000 |
| B2 | 0.000 | 1.400 | 0.000 | 2.200 | 0.000 | 1.100 | 0.000 | 1.800 | 1.400 | 0.000 | 0.000 | 1.600 | 1.700 | 1.800 |
| B3 | 0.000 | 0.000 | 0.000 | 0.000 | 1.100 | 0.000 | 1.700 | 2.000 | 0.000 | 0.000 | 0.000 | 0.000 | 0.000 | 0.000 |
| C1 | 0.000 | 2.000 | 1.800 | 2.700 | 1.500 | 1.700 | 0.000 | 2.200 | 2.000 | 1.400 | 0.000 | 2.000 | 0.000 | 2.400 |
| C2 | 0.000 | 1.600 | 1.200 | 2.700 | 0.000 | 0.000 | 2.100 | 0.000 | 1.600 | 1.300 | 0.000 | 1.700 | 1.600 | 0.000 |
| C3 | 0.000 | 2.000 | 0.000 | 1.700 | 1.300 | 0.000 | 0.000 | 0.000 | 0.000 | 0.000 | 0.000 | 0.000 | 0.000 | 0.000 |
| D1 | 1.600 | 0.000 | 1.700 | 2.600 | 1.800 | 1.900 | 0.000 | 0.000 | 0.900 | 0.000 | 1.800 | 1.800 | 1.700 | 1.800 |
| D2 | 0.000 | 0.000 | 1.800 | 2.200 | 0.000 | 1.300 | 0.000 | 0.000 | 0.000 | 1.700 | 0.000 | 1.000 | 1.300 | 1.300 |
| D3 | 0.000 | 1.700 | 2.300 | 2.100 | 1.500 | 1.300 | 0.000 | 1.600 | 0.000 | 1.800 | 1.200 | 0.000 | 2.000 | 2.100 |
| E1 | 0.000 | 0.000 | 2.400 | 1.800 | 1.600 | 0.000 | 1.900 | 1.500 | 0.000 | 1.800 | 1.500 | 2.000 | 0.000 | 2.100 |
| E2 | 0.000 | 0.000 | 2.000 | 2.400 | 1.600 | 0.000 | 2.300 | 1.500 | 0.000 | 1.700 | 0.000 | 2.100 | 0.000 | 0.000 |

**Table S6.** Normalized direct relation matrix.

|  | A1 | A2 | A3 | B1 | B2 | B3 | C1 | C2 | C3 | D1 | D2 | D3 | E1 | E2 |
| --- | --- | --- | --- | --- | --- | --- | --- | --- | --- | --- | --- | --- | --- | --- |
| A1 | 0.000 | 0.107 | 0.086 | 0.091 | 0.122 | 0.081 | 0.000 | 0.051 | 0.122 | 0.000 | 0.000 | 0.000 | 0.000 | 0.000 |
| A2 | 0.000 | 0.000 | 0.107 | 0.137 | 0.000 | 0.112 | 0.000 | 0.000 | 0.000 | 0.000 | 0.000 | 0.000 | 0.000 | 0.000 |
| A3 | 0.000 | 0.102 | 0.000 | 0.096 | 0.091 | 0.076 | 0.000 | 0.000 | 0.000 | 0.000 | 0.000 | 0.000 | 0.000 | 0.112 |
| B1 | 0.112 | 0.137 | 0.000 | 0.000 | 0.112 | 0.112 | 0.000 | 0.000 | 0.081 | 0.000 | 0.000 | 0.000 | 0.000 | 0.000 |
| B2 | 0.000 | 0.071 | 0.000 | 0.112 | 0.000 | 0.056 | 0.000 | 0.091 | 0.071 | 0.000 | 0.000 | 0.081 | 0.086 | 0.091 |
| B3 | 0.000 | 0.000 | 0.000 | 0.000 | 0.056 | 0.000 | 0.086 | 0.102 | 0.000 | 0.000 | 0.000 | 0.000 | 0.000 | 0.000 |
| C1 | 0.000 | 0.102 | 0.091 | 0.137 | 0.076 | 0.086 | 0.000 | 0.112 | 0.102 | 0.071 | 0.000 | 0.102 | 0.000 | 0.122 |
| C2 | 0.000 | 0.081 | 0.061 | 0.137 | 0.000 | 0.000 | 0.107 | 0.000 | 0.081 | 0.066 | 0.000 | 0.086 | 0.081 | 0.000 |
| C3 | 0.000 | 0.102 | 0.000 | 0.086 | 0.066 | 0.000 | 0.000 | 0.000 | 0.000 | 0.000 | 0.000 | 0.000 | 0.000 | 0.000 |
| D1 | 0.081 | 0.000 | 0.086 | 0.132 | 0.091 | 0.096 | 0.000 | 0.000 | 0.046 | 0.000 | 0.091 | 0.091 | 0.086 | 0.091 |
| D2 | 0.000 | 0.000 | 0.091 | 0.112 | 0.000 | 0.066 | 0.000 | 0.000 | 0.000 | 0.086 | 0.000 | 0.051 | 0.066 | 0.066 |
| D3 | 0.000 | 0.086 | 0.117 | 0.107 | 0.076 | 0.066 | 0.000 | 0.081 | 0.000 | 0.091 | 0.061 | 0.000 | 0.102 | 0.107 |
| E1 | 0.000 | 0.000 | 0.122 | 0.091 | 0.081 | 0.000 | 0.096 | 0.076 | 0.000 | 0.091 | 0.076 | 0.102 | 0.000 | 0.107 |
| E2 | 0.000 | 0.000 | 0.102 | 0.122 | 0.081 | 0.000 | 0.117 | 0.076 | 0.000 | 0.086 | 0.000 | 0.107 | 0.000 | 0.000 |

**Table S7.** Total influence matrix.

|  | A1 | A2 | A3 | B1 | B2 | B3 | C1 | C2 | C3 | D1 | D2 | D3 | E1 | E2 |
| --- | --- | --- | --- | --- | --- | --- | --- | --- | --- | --- | --- | --- | --- | --- |
| A1 | 0.024 | 0.196 | 0.133 | 0.203 | 0.193 | 0.156 | 0.032 | 0.098 | 0.167 | 0.019 | 0.006 | 0.037 | 0.030 | 0.046 |
| A2 | 0.020 | 0.050 | 0.123 | 0.178 | 0.050 | 0.154 | 0.020 | 0.027 | 0.025 | 0.007 | 0.002 | 0.013 | 0.009 | 0.024 |
| A3 | 0.023 | 0.160 | 0.050 | 0.185 | 0.151 | 0.138 | 0.036 | 0.049 | 0.037 | 0.025 | 0.007 | 0.041 | 0.024 | 0.145 |
| B1 | 0.123 | 0.201 | 0.048 | 0.094 | 0.169 | 0.173 | 0.026 | 0.048 | 0.123 | 0.013 | 0.005 | 0.028 | 0.023 | 0.031 |
| B2 | 0.033 | 0.169 | 0.091 | 0.254 | 0.100 | 0.140 | 0.059 | 0.156 | 0.124 | 0.055 | 0.024 | 0.144 | 0.129 | 0.153 |
| B3 | 0.010 | 0.051 | 0.039 | 0.074 | 0.092 | 0.038 | 0.111 | 0.135 | 0.037 | 0.026 | 0.007 | 0.040 | 0.026 | 0.036 |
| C1 | 0.050 | 0.249 | 0.207 | 0.346 | 0.219 | 0.218 | 0.072 | 0.201 | 0.181 | 0.134 | 0.029 | 0.187 | 0.068 | 0.215 |
| C2 | 0.042 | 0.199 | 0.158 | 0.295 | 0.119 | 0.113 | 0.146 | 0.069 | 0.145 | 0.116 | 0.029 | 0.151 | 0.124 | 0.088 |
| C3 | 0.015 | 0.135 | 0.023 | 0.129 | 0.092 | 0.040 | 0.008 | 0.017 | 0.021 | 0.005 | 0.002 | 0.013 | 0.011 | 0.015 |
| D1 | 0.122 | 0.134 | 0.197 | 0.315 | 0.231 | 0.216 | 0.065 | 0.096 | 0.120 | 0.067 | 0.119 | 0.172 | 0.145 | 0.190 |
| D2 | 0.036 | 0.081 | 0.162 | 0.227 | 0.098 | 0.146 | 0.044 | 0.057 | 0.045 | 0.126 | 0.026 | 0.106 | 0.102 | 0.134 |
| D3 | 0.047 | 0.209 | 0.235 | 0.307 | 0.211 | 0.191 | 0.074 | 0.167 | 0.074 | 0.158 | 0.094 | 0.097 | 0.163 | 0.209 |
| E1 | 0.048 | 0.145 | 0.247 | 0.308 | 0.225 | 0.137 | 0.160 | 0.170 | 0.085 | 0.169 | 0.110 | 0.204 | 0.076 | 0.227 |
| E2 | 0.045 | 0.133 | 0.198 | 0.296 | 0.202 | 0.120 | 0.161 | 0.155 | 0.079 | 0.142 | 0.029 | 0.184 | 0.063 | 0.101 |

**Table S8.** Degree of influence.

|  | **R** | **C** | **R+C** | **R-C** | **Influence** |
| --- | --- | --- | --- | --- | --- |
| A1 | 1.341 | 0.638 | 1.979 | 0.703 | Cause |
| A2 | 0.702 | 2.113 | 2.814 | -1.411 | Effect |
| A3 | 1.072 | 1.910 | 2.982 | -0.838 | Effect |
| B1 | 1.105 | 3.212 | 4.317 | -2.107 | Effect |
| B2 | 1.630 | 2.151 | 3.782 | -0.521 | Effect |
| B3 | 0.722 | 1.979 | 2.701 | -1.257 | Effect |
| C1 | 2.376 | 1.015 | 3.391 | 1.361 | Cause |
| C2 | 1.795 | 1.446 | 3.241 | 0.350 | Cause |
| C3 | 0.528 | 1.263 | 1.791 | -0.735 | Effect |
| D1 | 2.188 | 1.061 | 3.249 | 1.127 | Cause |
| D2 | 1.388 | 0.487 | 1.875 | 0.900 | Cause |
| D3 | 2.237 | 1.416 | 3.653 | 0.820 | Cause |
| E1 | 2.308 | 0.992 | 3.300 | 1.316 | Cause |
| E2 | 1.907 | 1.615 | 3.522 | 0.293 | Cause |
